# (2-{[2-(2-Amino­ethyl­amino)­eth­yl]imino­meth­yl}phenolato)nickel(II) chloride dihydrate

**DOI:** 10.1107/S1600536810050026

**Published:** 2010-12-04

**Authors:** Dong’e Wang

**Affiliations:** a Department of Chemistry, Kashgar Teachers College, Kashgar 844000, People’s Republic of China

## Abstract

In the title complex, [Ni(C_11_H_16_N_3_O)]Cl·2H_2_O, the Ni^II^ ion is coordinated within a distorted square-planar environment. In the crystal, inter­molecular N—H⋯Cl, N—H⋯O, O—H⋯O, O—H⋯Cl and weak C—H⋯O hydrogen bonds link the components into a two-dimensional network parallel to (001).

## Related literature

For related structures, see: Chen & Wang (2006 ▶); Cusmano Priolo *et al.* (1983 ▶); Kratochvíl *et al.* (1989 ▶, 1991 ▶); Liu *et al.* (2004 ▶); Loub *et al.* (1989 ▶, 1990 ▶); Podlahová *et al.* (1988 ▶); Rotondo *et al.* (1983 ▶); Zhang *et al.* (2006 ▶); Zhu *et al.* (2004 ▶).
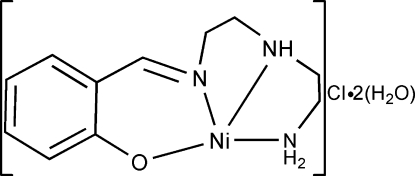


## Experimental

### 

#### Crystal data

[Ni(C_11_H_16_N_3_O)]Cl·2H_2_O
*M* *_r_* = 336.46
Monoclinic, 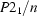

*a* = 7.1062 (16) Å
*b* = 11.6685 (19) Å
*c* = 17.677 (2) Å
β = 96.699 (3)°
*V* = 1455.8 (4) Å^3^
*Z* = 4
Mo *K*α radiation
μ = 1.52 mm^−1^
*T* = 298 K
0.20 × 0.06 × 0.04 mm

#### Data collection

Bruker SMART APEX I CCD area-detector diffractometer
Absorption correction: multi-scan (*SADABS*; Sheldrick, 1996) ▶ *T*_min_ = 0.783, *T*_max_ = 0.863
13581 measured reflections
2565 independent reflections
1860 reflections with *I* > 2σ(*I*)
*R*_int_ = 0.099

#### Refinement

*R*[*F* ^2^ > 2σ(*F* ^2^)] = 0.079
*wR*(*F* ^2^) = 0.152
*S* = 1.16
2565 reflections
193 parameters
10 restraints
H atoms treated by a mixture of independent and constrained refinement
Δρ_max_ = 0.41 e Å^−3^
Δρ_min_ = −0.67 e Å^−3^

### 

Data collection: *SMART* (Bruker, 2001 ▶); cell refinement: *SAINT-Plus* (Bruker, 2001 ▶); data reduction: *SAINT-Plus*; program(s) used to solve structure: *SHELXS97* (Sheldrick, 2008 ▶); program(s) used to refine structure: *SHELXL97* (Sheldrick, 2008 ▶); molecular graphics: *SHELXTL* (Sheldrick, 2008 ▶); software used to prepare material for publication: *SHELXTL*.

## Supplementary Material

Crystal structure: contains datablocks global, I. DOI: 10.1107/S1600536810050026/lh5172sup1.cif

Structure factors: contains datablocks I. DOI: 10.1107/S1600536810050026/lh5172Isup2.hkl

Additional supplementary materials:  crystallographic information; 3D view; checkCIF report

## Figures and Tables

**Table 1:** Hydrogen-bond geometry (Å, °)

*D*—H⋯*A*	*D*—H	H⋯*A*	*D*⋯*A*	*D*—H⋯*A*
N2—H2*A*⋯Cl1^i^	0.87 (2)	2.55 (4)	3.325 (6)	149 (6)
N3—H3*A*⋯Cl1	0.84 (4)	2.60 (3)	3.397 (6)	160 (5)
N3—H3*B*⋯O3^ii^	0.85 (2)	2.09 (3)	2.914 (8)	162 (6)
O2—H2*B*⋯Cl1	0.83 (2)	2.27 (5)	3.091 (5)	176 (9)
O2—H2*C*⋯O1	0.82 (6)	1.99 (6)	2.797 (6)	172 (8)
O3—H3*C*⋯O2	0.82 (2)	1.93 (2)	2.750 (8)	175 (9)
O3—H3*D*⋯Cl1^iii^	0.81 (7)	2.36 (7)	3.166 (6)	172 (9)
C25—H25*B*⋯O2^i^	0.97	2.56	3.464 (9)	154

Symmetry codes: (i) 
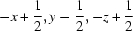
; (ii) 
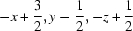
; (iii) 
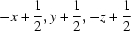
.

**Fig. 1.**
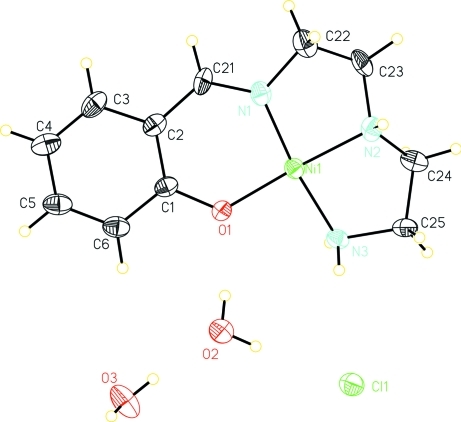
Molecular structure of (I), showing the atom-numbering scheme. Displacement ellipsoids are drawn at the 50% probability level.

**Fig. 2.**
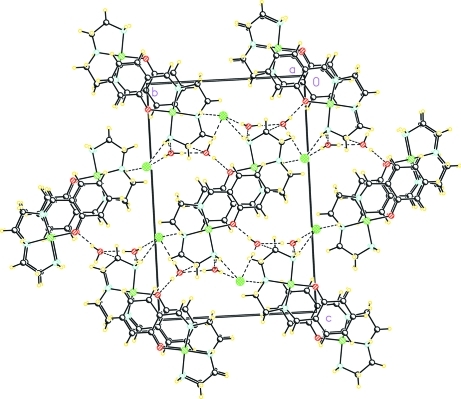
Part of the crystal structure of (I), showing the formation of the two-dimensional network parallel to the (001) plane. Hydrogen bonds are shown as dashed lines.

## supplementary crystallographic information

### Comment

The Schiff base ligand 2-((2-(2-aminoethylamino)ethylimino)methyl)phenol has often been used in the synthesis of metal-organic complexes (Chen & Wang, 2006, Cusmano Priolo *et al.*, 1983, Kratochvíl *et al.*, 1989, Kratochvíl *et al.*,1991, Loub *et al.*, 1990, Loub *et al.*, 1989, Podlahová *et al.*, 1988, Rotondo *et al.*, 1983, Zhang *et al.*, 2006, Zhu *et al.*,2004, Liu *et al.*, 2004). In this paper, we report the title mononuclear metal complex (I).

In (I), the asymmetric unit consists of a coordination cation, one uncoordinated Cl^-^ anion and two solvent water molecules (Fig.1). The Ni^II^ ion is in a distorted square-planar coordination environmemt with atom Ni1 atom 0.058Å from the plane formed by N1/N2/N3/O1. The Ni—N/O bond lengths are comparable to previously published analogs (Loub *et al.*, 1989, Podlahová *et al.*, 1988).

The crystal structure is stabilized by intermolecular hydrogen bonds (Table 1), forming a two-dimensional network parallel to the (001) plane (Fig.2).

### Experimental

NiCl_2_.6(H_2_O) (1 mmol, 238 mg), salicylaldehyde (1 mmol, 122 mg) and diethylenetriamine (1 mmol, 103 mg) were dissolved in a mixture of ethanol and acetonitrile (50 ml, 1:1 *v*/*v*), resulting in a light-green solution. When diethyl ether was slowly diffused into this solution for one week, pale-yellow blocks suitable for X-ray diffraction were formed at the bottom of the vessel.

### Refinement

All the H atoms bonded to carbon atoms were located at their geometrical positions with C–H = 0.97 Å(methylene) and 0.93 Å(aromatic), *U*_iso_(H) = 1.2*U*_eq_(C). H atoms bonded to imine N and water O atoms were located on the difference Fourier maps and then refined with the constraints of N—H = 0.86 (2) Å, O—H = 0.82 (2) Å, H—H = 1.35 (2)Å and the *U*_iso_(H) values were set 1.2 times of *U*_eq_(N) or 1.5 times of *U*_eq_(O) of their carrier atoms.

### Crystal data

**Table:**

[Ni(C_11_H_16_N_3_O)]Cl·2H_2_O	*F*(000) = 704
*M**_r_* = 336.46	*D*_x_ = 1.535 Mg m^−^^3^
Monoclinic, *P*2_1_/*n*	Mo *K*α radiation, λ = 0.71073 Å
Hall symbol: -P 2yn	Cell parameters from 1489 reflections
*a* = 7.1062 (16) Å	θ = 1.7–19.5°
*b* = 11.6685 (19) Å	µ = 1.52 mm^−^^1^
*c* = 17.677 (2) Å	*T* = 298 K
β = 96.699 (3)°	Needle, yellow
*V* = 1455.8 (4) Å^3^	0.20 × 0.06 × 0.04 mm
*Z* = 4	

### Data collection

**Table:**

Bruker SMART APEX I CCD area-detector diffractometer	2565 independent reflections
Radiation source: fine focus sealed Siemens Mo tube	1860 reflections with *I* > 2σ(*I*)
graphite	*R*_int_ = 0.099
0.3° wide ω exposures scans	θ_max_ = 25.0°, θ_min_ = 2.1°
Absorption correction: multi-scan (*SADABS*; Sheldrick, 1996)	*h* = −8→8
*T*_min_ = 0.783, *T*_max_ = 0.863	*k* = −13→13
13581 measured reflections	*l* = −21→21

### Refinement

**Table:**

Refinement on *F*^2^	Primary atom site location: structure-invariant direct methods
Least-squares matrix: full	Secondary atom site location: difference Fourier map
*R*[*F*^2^ > 2σ(*F*^2^)] = 0.079	Hydrogen site location: inferred from neighbouring sites
*wR*(*F*^2^) = 0.152	H atoms treated by a mixture of independent and constrained refinement
*S* = 1.16	*w* = 1/[σ^2^(*F*_o_^2^) + (0.0551*P*)^2^] where *P* = (*F*_o_^2^ + 2*F*_c_^2^)/3
2565 reflections	(Δ/σ)_max_ < 0.001
193 parameters	Δρ_max_ = 0.41 e Å^−^^3^
10 restraints	Δρ_min_ = −0.67 e Å^−^^3^

### Special details

**Table:**

Geometry. All e.s.d.'s (except the e.s.d. in the dihedral angle between two l.s. planes) are estimated using the full covariance matrix. The cell e.s.d.'s are taken into account individually in the estimation of e.s.d.'s in distances, angles and torsion angles; correlations between e.s.d.'s in cell parameters are only used when they are defined by crystal symmetry. An approximate (isotropic) treatment of cell e.s.d.'s is used for estimating e.s.d.'s involving l.s. planes.
Refinement. Refinement of *F*^2^ against ALL reflections. The weighted *R*-factor *wR* and goodness of fit *S* are based on *F*^2^, conventional *R*-factors *R* are based on *F*, with *F* set to zero for negative *F*^2^. The threshold expression of *F*^2^ > σ(*F*^2^) is used only for calculating *R*-factors(gt) *etc*. and is not relevant to the choice of reflections for refinement. *R*-factors based on *F*^2^ are statistically about twice as large as those based on *F*, and *R*- factors based on ALL data will be even larger.

### Fractional atomic coordinates and isotropic or equivalent isotropic displacement parameters (Å^2^)

**Table:**

	*x*	*y*	*z*	*U*_iso_*/*U*_eq_	
Ni1	0.63623 (11)	0.34513 (7)	0.37464 (5)	0.0333 (3)	
C1	0.7236 (9)	0.5502 (6)	0.4572 (4)	0.0358 (17)	
C2	0.7687 (9)	0.4916 (6)	0.5255 (4)	0.0385 (17)	
C3	0.8289 (10)	0.5530 (7)	0.5919 (4)	0.051 (2)	
H3	0.8572	0.5139	0.6376	0.061*	
C4	0.8469 (10)	0.6701 (8)	0.5906 (5)	0.056 (2)	
H4	0.8896	0.7100	0.6348	0.067*	
C5	0.8020 (10)	0.7266 (7)	0.5245 (4)	0.051 (2)	
H5	0.8110	0.8061	0.5242	0.061*	
C6	0.7432 (9)	0.6705 (6)	0.4571 (4)	0.0454 (19)	
H6	0.7166	0.7117	0.4121	0.055*	
C21	0.7525 (9)	0.3699 (6)	0.5302 (4)	0.0416 (18)	
H21	0.7820	0.3364	0.5778	0.050*	
C22	0.6905 (10)	0.1786 (6)	0.4877 (5)	0.053 (2)	
H22A	0.5692	0.1585	0.5041	0.064*	
H22B	0.7903	0.1556	0.5268	0.064*	
C23	0.7149 (10)	0.1201 (6)	0.4129 (4)	0.048 (2)	
H23A	0.8472	0.1201	0.4042	0.058*	
H23B	0.6708	0.0415	0.4133	0.058*	
C24	0.6345 (10)	0.1642 (6)	0.2747 (4)	0.0479 (19)	
H24A	0.5831	0.0902	0.2580	0.058*	
H24B	0.7693	0.1647	0.2704	0.058*	
C25	0.5361 (10)	0.2590 (5)	0.2279 (4)	0.0427 (19)	
H25A	0.5694	0.2569	0.1763	0.051*	
H25B	0.3997	0.2515	0.2261	0.051*	
Cl1	0.2875 (3)	0.52634 (16)	0.14998 (11)	0.0494 (5)	
N1	0.7000 (7)	0.3025 (5)	0.4737 (3)	0.0395 (14)	
N2	0.5992 (8)	0.1873 (5)	0.3525 (3)	0.0426 (15)	
H2A	0.488 (5)	0.167 (6)	0.364 (4)	0.051*	
N3	0.6022 (8)	0.3689 (4)	0.2664 (3)	0.0357 (14)	
H3A	0.507 (4)	0.405 (5)	0.247 (3)	0.043*	
H3B	0.711 (4)	0.384 (5)	0.253 (3)	0.043*	
O1	0.6663 (6)	0.5003 (4)	0.3910 (2)	0.0374 (11)	
O2	0.4176 (8)	0.6467 (4)	0.3029 (3)	0.0581 (14)	
H2B	0.386 (12)	0.617 (6)	0.261 (2)	0.087*	
H2C	0.484 (11)	0.599 (5)	0.327 (3)	0.087*	
O3	0.5374 (8)	0.8703 (5)	0.2971 (4)	0.0741 (18)	
H3C	0.508 (12)	0.803 (2)	0.300 (6)	0.111*	
H3D	0.446 (8)	0.906 (6)	0.308 (6)	0.111*	

### Atomic displacement parameters (Å^2^)

**Table:**

	*U*^11^	*U*^22^	*U*^33^	*U*^12^	*U*^13^	*U*^23^
Ni1	0.0315 (5)	0.0318 (5)	0.0368 (5)	0.0024 (4)	0.0045 (4)	0.0000 (4)
C1	0.028 (4)	0.046 (5)	0.033 (4)	−0.002 (3)	0.003 (3)	−0.005 (4)
C2	0.029 (4)	0.053 (5)	0.034 (4)	0.005 (3)	0.004 (3)	−0.002 (4)
C3	0.036 (4)	0.076 (6)	0.038 (5)	0.010 (4)	−0.002 (4)	0.002 (4)
C4	0.048 (5)	0.071 (7)	0.047 (5)	−0.008 (4)	−0.002 (4)	−0.014 (5)
C5	0.046 (5)	0.055 (5)	0.051 (6)	0.001 (4)	0.010 (4)	−0.020 (4)
C6	0.036 (4)	0.049 (5)	0.051 (5)	−0.004 (4)	0.004 (3)	−0.013 (4)
C21	0.037 (4)	0.054 (5)	0.034 (4)	0.015 (4)	0.005 (3)	0.014 (4)
C22	0.037 (4)	0.045 (5)	0.076 (6)	−0.005 (4)	0.003 (4)	0.007 (4)
C23	0.040 (4)	0.027 (4)	0.078 (6)	0.009 (3)	0.006 (4)	0.004 (4)
C24	0.045 (4)	0.043 (4)	0.057 (5)	−0.005 (4)	0.012 (4)	−0.015 (4)
C25	0.047 (5)	0.043 (5)	0.038 (4)	−0.009 (4)	0.003 (4)	−0.011 (3)
Cl1	0.0508 (12)	0.0435 (11)	0.0529 (12)	0.0020 (9)	0.0017 (10)	0.0043 (9)
N1	0.032 (3)	0.038 (3)	0.049 (4)	0.008 (3)	0.005 (3)	0.004 (3)
N2	0.041 (4)	0.042 (4)	0.045 (4)	−0.010 (3)	0.007 (3)	0.012 (3)
N3	0.030 (3)	0.032 (4)	0.044 (4)	0.001 (3)	0.003 (3)	−0.007 (3)
O1	0.042 (3)	0.035 (3)	0.033 (3)	0.003 (2)	−0.003 (2)	0.000 (2)
O2	0.071 (4)	0.046 (3)	0.056 (3)	0.009 (3)	−0.001 (3)	−0.002 (3)
O3	0.066 (4)	0.042 (3)	0.118 (5)	0.003 (3)	0.027 (4)	−0.004 (4)

### Geometric parameters (Å, °)

**Table:**

Ni1—N1	1.825 (6)	C22—H22A	0.9700
Ni1—O1	1.842 (4)	C22—H22B	0.9700
Ni1—N2	1.894 (6)	C23—N2	1.492 (9)
Ni1—N3	1.920 (6)	C23—H23A	0.9700
C1—O1	1.329 (7)	C23—H23B	0.9700
C1—C2	1.392 (9)	C24—N2	1.452 (9)
C1—C6	1.410 (9)	C24—C25	1.504 (9)
C2—C3	1.398 (10)	C24—H24A	0.9700
C2—C21	1.429 (9)	C24—H24B	0.9700
C3—C4	1.373 (10)	C25—N3	1.501 (8)
C3—H3	0.9300	C25—H25A	0.9700
C4—C5	1.346 (10)	C25—H25B	0.9700
C4—H4	0.9300	N2—H2A	0.87 (2)
C5—C6	1.381 (9)	N3—H3A	0.84 (4)
C5—H5	0.9300	N3—H3B	0.854 (19)
C6—H6	0.9300	O2—H2B	0.83 (2)
C21—N1	1.292 (8)	O2—H2C	0.82 (6)
C21—H21	0.9300	O3—H3C	0.82 (2)
C22—N1	1.469 (8)	O3—H3D	0.81 (7)
C22—C23	1.515 (10)		
			
N1—Ni1—O1	96.1 (2)	C22—C23—H23A	110.5
N1—Ni1—N2	86.9 (3)	N2—C23—H23B	110.5
O1—Ni1—N2	176.9 (2)	C22—C23—H23B	110.5
N1—Ni1—N3	169.4 (2)	H23A—C23—H23B	108.7
O1—Ni1—N3	90.7 (2)	N2—C24—C25	105.3 (6)
N2—Ni1—N3	86.4 (2)	N2—C24—H24A	110.7
O1—C1—C2	124.4 (6)	C25—C24—H24A	110.7
O1—C1—C6	117.1 (6)	N2—C24—H24B	110.7
C2—C1—C6	118.5 (6)	C25—C24—H24B	110.7
C1—C2—C3	119.5 (7)	H24A—C24—H24B	108.8
C1—C2—C21	121.8 (6)	N3—C25—C24	106.1 (5)
C3—C2—C21	118.6 (7)	N3—C25—H25A	110.5
C4—C3—C2	121.0 (7)	C24—C25—H25A	110.5
C4—C3—H3	119.5	N3—C25—H25B	110.5
C2—C3—H3	119.5	C24—C25—H25B	110.5
C5—C4—C3	119.2 (7)	H25A—C25—H25B	108.7
C5—C4—H4	120.4	C21—N1—C22	118.9 (6)
C3—C4—H4	120.4	C21—N1—Ni1	126.3 (5)
C4—C5—C6	122.3 (8)	C22—N1—Ni1	114.7 (5)
C4—C5—H5	118.9	C24—N2—C23	116.0 (6)
C6—C5—H5	118.9	C24—N2—Ni1	109.9 (4)
C5—C6—C1	119.3 (7)	C23—N2—Ni1	108.2 (4)
C5—C6—H6	120.3	C24—N2—H2A	116 (5)
C1—C6—H6	120.3	C23—N2—H2A	97 (5)
N1—C21—C2	125.4 (6)	Ni1—N2—H2A	109 (5)
N1—C21—H21	117.3	C25—N3—Ni1	109.0 (4)
C2—C21—H21	117.3	C25—N3—H3A	93 (4)
N1—C22—C23	106.5 (6)	Ni1—N3—H3A	119 (4)
N1—C22—H22A	110.4	C25—N3—H3B	107 (4)
C23—C22—H22A	110.4	Ni1—N3—H3B	107 (4)
N1—C22—H22B	110.4	H3A—N3—H3B	120 (4)
C23—C22—H22B	110.4	C1—O1—Ni1	126.0 (4)
H22A—C22—H22B	108.6	H2B—O2—H2C	104 (3)
N2—C23—C22	106.1 (5)	H3C—O3—H3D	105 (8)
N2—C23—H23A	110.5		
			
O1—C1—C2—C3	179.6 (6)	N3—Ni1—N1—C21	128.3 (13)
C6—C1—C2—C3	1.0 (9)	O1—Ni1—N1—C22	178.7 (4)
O1—C1—C2—C21	−1.4 (10)	N2—Ni1—N1—C22	−0.8 (5)
C6—C1—C2—C21	179.9 (6)	N3—Ni1—N1—C22	−51.1 (15)
C1—C2—C3—C4	−1.0 (10)	C25—C24—N2—C23	−167.6 (5)
C21—C2—C3—C4	−180.0 (6)	C25—C24—N2—Ni1	−44.5 (6)
C2—C3—C4—C5	1.4 (11)	C22—C23—N2—C24	166.1 (6)
C3—C4—C5—C6	−1.8 (11)	C22—C23—N2—Ni1	42.1 (6)
C4—C5—C6—C1	1.9 (11)	N1—Ni1—N2—C24	−151.6 (5)
O1—C1—C6—C5	179.9 (6)	N3—Ni1—N2—C24	20.3 (5)
C2—C1—C6—C5	−1.4 (10)	N1—Ni1—N2—C23	−24.0 (4)
C1—C2—C21—N1	1.5 (11)	N3—Ni1—N2—C23	147.9 (5)
C3—C2—C21—N1	−179.5 (6)	C24—C25—N3—Ni1	−35.4 (6)
N1—C22—C23—N2	−42.0 (7)	N1—Ni1—N3—C25	59.6 (15)
N2—C24—C25—N3	51.1 (7)	O1—Ni1—N3—C25	−169.8 (4)
C2—C21—N1—C22	179.9 (6)	N2—Ni1—N3—C25	9.2 (4)
C2—C21—N1—Ni1	0.6 (10)	C2—C1—O1—Ni1	−0.6 (9)
C23—C22—N1—C21	−154.8 (6)	C6—C1—O1—Ni1	178.0 (4)
C23—C22—N1—Ni1	24.7 (7)	N1—Ni1—O1—C1	2.0 (5)
O1—Ni1—N1—C21	−1.9 (6)	N3—Ni1—O1—C1	−170.0 (5)
N2—Ni1—N1—C21	178.6 (6)		

### Hydrogen-bond geometry (Å, °)

**Table:**

*D*—H···*A*	*D*—H	H···*A*	*D*···*A*	*D*—H···*A*
N2—H2A···Cl1^i^	0.87 (2)	2.55 (4)	3.325 (6)	149 (6)
N3—H3A···Cl1	0.84 (4)	2.60 (3)	3.397 (6)	160 (5)
N3—H3B···O3^ii^	0.85 (2)	2.09 (3)	2.914 (8)	162 (6)
O2—H2B···Cl1	0.83 (2)	2.27 (5)	3.091 (5)	176 (9)
O2—H2C···O1	0.82 (6)	1.99 (6)	2.797 (6)	172 (8)
O3—H3C···O2	0.82 (2)	1.93 (2)	2.750 (8)	175 (9)
O3—H3D···Cl1^iii^	0.81 (7)	2.36 (7)	3.166 (6)	172 (9)
C25—H25B···O2^i^	0.97	2.56	3.464 (9)	154.

Symmetry codes: (i) −*x*+1/2, *y*−1/2, −*z*+1/2; (ii) −*x*+3/2, *y*−1/2, −*z*+1/2; (iii) −*x*+1/2, *y*+1/2, −*z*+1/2.

## References

[bb1] Bruker (2001). *SAINT-Plus* and *SMART* Bruker AXS Inc., Madison, Wisconsin, USA.

[bb2] Chen, K. & Wang, J.-H. (2006). *Acta Cryst.* E**62**, m2305–m2306.

[bb3] Cusmano Priolo, F., Rotondo, E., Rizzardi, G., Bruno, G. & Bombieri, G. (1983). *Acta Cryst.* C**39**, 550–552.

[bb4] Kratochvíl, B., Nováková, M., Haber, V., Ondráček, J. & Hájek, B. (1989). *Acta Cryst.* C**45**, 403–405.

[bb5] Kratochvíl, B., Ondráček, J., Novotný, J. & Haber, V. (1991). *Acta Cryst.* C**47**, 2207–2209.

[bb6] Liu, G. X., Ren, X. M., Xu, H., Tang, C. Y., Wu, G. H. & Chen, Y. C. (2004). *Chin. Chem. Lett.* **15**, 1105–1108.

[bb7] Loub, J., Podlahová, J., Haber, V., Kopf, J. & Weiss, E. (1990). *Acta Cryst.* C**46**, 596–598.

[bb8] Loub, J., Podlahová, J., Kopf, J. & Weiss, E. (1989). *Acta Cryst.* C**45**, 406–407.

[bb9] Podlahová, J., Knížek, K., Loub, J. & Hašek, J. (1988). *Acta Cryst.* C**44**, 631–633.

[bb10] Rotondo, E., Cusmano Priolo, F., Romeo, M., Bruno, G. & Bombieri, G. (1983). *Acta Cryst.* C**39**, 1525–1527.

[bb11] Sheldrick, G. M. (1996). *SADABS* Bruker AXS Inc., Madison, Wisconsin, USA.

[bb12] Sheldrick, G. M. (2008). *Acta Cryst.* A**64**, 112–122. 10.1107/S0108767307043930 18156677

[bb13] Zhang, H.-W., Hu, S., Zhang, L.-N. & Fang, R.-Q. (2006). *Acta Cryst.* E**62**, m1275–m1277.

[bb14] Zhu, H. L., Li, S. Y., Wang, Z. D. & Yang, F. (2004). *J. Chem. Crystallogr.* **34**, 203–206.

